# Supplementary material for: Views of patients suffering from Failed Back Surgery Syndrome on their health and their ability to adapt to daily life and self-management: A qualitative exploration
Source: PLoS One. 2020 Dec 7;15(12):e0243329. doi: 10.1371/journal.pone.0243329 (PMC7721158; doi:10.1371/journal.pone.0243329)
Supplement: S2 File — (DOCX) [file pone.0243329.s002.docx]

Topic list

1. Welcome
2. Can you describe the pain in your body?
   1. Do you know the cause of your pain problem?
   2. What are your daily limitations, and how do you manage this?
   3. What treatments did you have for your pain?
   4. In what way does pain medication influence your activities?
   5. How does chronic pain affect your appetite, and what are the consequences for you?
   6. What do you like to do and is not possible due to chronic pain?
   7. How do you handle this?
   8. Can you say something about your quality of sleep and the consequences?
   9. How does chronic pain affect your energy level?
3. How does chronic pain influence your mental health status/ mood?
   1. De you experience feelings of depression?
   2. Do you experience feelings of anxiety?
   3. How do you control those feelings/ professional help?
   4. How does chronic pain affect your self-control?
   5. What does this mentally do to you?
   6. What do you do to relax?
   7. How do you handle changes in your life?
4. How do you deal with the most essential things in your life?
   1. Do you have a goal in your life?
   2. How do you want to accomplish this goal, living with chronic pain?
   3. How do your partner, family, friends support you?
   4. What are your wishes and dreams for the future?
   5. What is realistic for you?
   6. What did you learn about yourself living with chronic pain/ limitations?
   7. Do you accept your situation? What should change?
5. Can you describe your Quality of Life?
   1. What changed in your quality of life since you have chronic pain?
   2. How do you describe your health status?
   3. What means happiness to you?
   4. Where/ how do you find happiness in your life?
   5. How do you wish to express yourself to the outside world?
   6. What do you need for this/ what could society do?
   7. Are you open for a question about sexuality?
   8. What is quality time for you?
6. How do you combine chronic pain with your social life?
   1. Could you tell something about your current social life?
   2. How important is work for you?
   3. How do you balance your work with chronic pain?
   4. What are the consequences of not having a paid job/ financial?
   5. How are you supported by society or (care) professionals or family environment?
   6. Are you a member of a social club?
   7. Do you feel isolated?
7. How do you manage your daily activities?
   1. Can you describe your day?
   2. What is the best part of the day?
   3. What are physical or emotional obstacles during the day?
   4. How do you handle this?
   5. What do you need to balance your life?
   6. What do you need for the future?
8. Is there something else you want to say before we end this interview?
9. Thank the participant.
