# Supplementary material for: Views of patients suffering from Failed Back Surgery Syndrome on their health and their ability to adapt to daily life and self-management: A qualitative exploration
Source: PLoS One. 2020 Dec 7;15(12):e0243329. doi: 10.1371/journal.pone.0243329 (PMC7721158; doi:10.1371/journal.pone.0243329)
Supplement: S4 File — (DOCX) [file pone.0243329.s004.docx]

COREQ (COnsolidated criteria for REporting Qualitative research) Checklist A checklist of items that should be included in reports of qualitative research. You must report the page number in your manuscript where you consider each of the items listed in this checklist. If you have not included this information, either revise your manuscript accordingly before submitting or note N/A.

| **Topic** | **Item No.** | **Guide Questions/Description** | **Reported on Page No.** |
| --- | --- | --- | --- |
| **Domain 1: Research team and reflexivity** |  |  |  |
| *Personal characteristics* |  |  |  |
| Interviewer/facilitator | 1 | Which author/s conducted the interview or focus group? | **Page 4,**  T.H. |
| Credentials | 2 | What were the researcher’s credentials?  E.g. PhD, MD | **Page 4,**  MA-ANP |
| Occupation | 3 | What was their occupation at the time of the study? | **Page 4,**  Registered Nurse Practitioner |
| Gender | 4 | Was the researcher male or female | **Page 4,**  Female |
| Experience and training | 5 | What experience or training did the researcher have? | **Page 4,**  She followed a qualitative certificated research training by IQ HealthCare. Furthermore, she was trained in communication technics by discussing audio recorded interviews with an experienced interviewer, and received feedback from two researches experienced in qualitative research. |
| Relationship with participants |  |  |  |
| Relationship established | 6 | Was a relationship established prior to study commencement? | **Page 4,**  No |
| Participant knowledge of the interviewer | 7 | What did the participants know about the researcher? e.g. personal goals, reasons for doing the research | **Page 4,**  That the researcher wants to do interviews to explore the QoL of FBSS participants in the period before the receive an SCS, and that she works at the department of pain medicine. |
| Interviewer characteristics | 8 | What characteristics were reported about the inter viewer/facilitator? e.g. Bias, assumptions, reasons and interests in the research topic | **Page 4,**  The interviewer is a nurse practitioner at the department of pain medicine. She is specialized in management of chronic pain and in the field of Neuromodulation. She in trained in qualitative research and communication techniques.  She had no personal or professional history with the participants |
| **Domain 2: Study design** |  |  |  |
| *Theoretical framework* |  |  |  |
| Methodological orientation and Theory | 9 | What methodological orientation was stated to underpin the study? e.g. grounded theory, discourse analysis, ethnography, phenomenology, content analysis | **Page 8,**  For the qualitative content analysis, we used the phases of thematic analysis with an inductive approach using the six dimensions of Positive Health as themes for the semi-structured in-depth interviews. (ref: Braun Virginia et al. Using thematic analysis in psychology, Qualitative research in psychology, January 2006, 3(2):77-101).  The interviews were audio recorded and transcribed verbatim, after which it was coded line by line by two independent researches until consensus was reached, using ATLAS.ti scientific software |
| *Participant selection* |  |  |  |
| Sampling | 10 | How were participants selected? e.g. purposive, convenience, consecutive, snowball | **Page 5,**  We did purposive sampling to collect participants. |
| Method of approach | 11 | How were participants approached? e.g. face-to-face, telephone, mail, email | **Page 6,**  We did a face-to-face interview |
| Sample size | 12 | How many participants were in the study? | **Page 9,**  In total 17 participants were included. We had saturation after 15 interviews and did two more interviews to confirm saturation. |
| Non-participation | 13 | How many people refused to participate or dropped out? Reasons? | **Page 9,**  None of the participants refused their participation for this study. |
| *Setting* |  |  |  |
| Setting of data collection | 14 | Where was the data collected? e.g. home, clinic, workplace Presence of nonparticipant | **Page 5,**  At a comfortable environment in the hospital.  This location was explained in the manuscript. |
| Presence of nonparticipants | 15 | Was anyone else present besides the participants and researchers? | **Page 9,**  During the interview, only the participant and the interviewer were present |
| Description of sample | 16 | What are the important characteristics of the sample? e.g. demographic data, date | **Page 5,**  A representative distribution of Dutch men and women between 18- 75 years, suffering low back pain and leg after surgery on the Spine, at the department of pain medicine. Furthermore, participants had to meet the inclusion criteria of the Dutch Neuromodulation Society. We included until saturation was reached, after which we did two more interviews to confirm saturation. |
| *Data collection* |  |  |  |
| Interview guide | 17 | Were questions, prompts, guides provided by the authors? Was it pilot tested? | **Page 7,**  We used a  topic list guided by the six dimensions of Positive Health and a Web diagram of the six dimension of Positive Health which participants filled in at the start of the interview. |
| Repeat interviews | 18 | Were repeat interviews carried out? If yes, how many? | **Pag 8,**  it was not needed to repeat any of the interviews, |
| Audio/visual recording | 19 | Did the research use audio or visual recording to collect the data? | **Pag 7,**  The interviews were audio-recorded and transcribed verbatim. |
| Field notes | 20 | Were field notes made during and/or after the interview or focus group? | **Pag 8,**  Field notes were made for elements which cannot be recorded |
| Duration | 21 | What was the duration of the interviews or focus group? | **Page 9,**  Each interview lasted between 45-60 minutes |
| Data saturation | 22 | Was data saturation discussed? | **Page 8,**  Two researchers (T.H. and D.H.), who met periodically to compare individual coding and to discuss codes and themes until consensus was reached. |
| Transcripts returned | 23 | Were transcripts returned to participants for comment and/or correction | **Pag 8,**  The transcripts were not returned to the participants for their comments. |
| **Domain: analysis and findings** |  |  |  |
| *Data analysis* |  |  |  |
| Number of data codes | 24 | How many data coders coded the data? | **Page 9,**  These themes arose from eleven categories and 190 codes |
| Description of the coding tree | 25 | Did authors provide a description of the coding tree? | **Pag 7,**  The coding tree is added as a supplementary file 1.  Starting after the first interview had taken place, transcriptions were coded line-by-line, through which a code list was created. The code list derived from the previous interview was used as a starting point for coding the next one. Coding was continued after each interview.  Coding and analysis were carried out independently by two researchers |
| Derivation of themes | 26 | Were themes identified in advance or derived from the data? | **Pag 9,**  After having analysed the interviews, three themes emerged |
| Software | 27 | What software, if applicable, was used to manage the data? | **Pag 8,**  The coding was performed using ATLAS.ti. Scientific Software |
| Participant checking | 28 | Did participants provide feedback on the findings? | **Pag 8,**  The transcripts were not returned to the participants for their comments. |
| *Reporting* |  |  |  |
| Quotations presented | 29 | Were participant quotations presented to illustrate the themes/findings? Was each quotation identified? e.g. participant number | **Page 9-19,**  In the results section  With a participant number |
| Data and findings consistent | 30 | Was there consistency between the data presented and the findings? | **Page 9,**  The data presented the views of patients suffering chronic pain, on their health and their ability to adapt to daily life and self-management. The data presented the shortcoming of these participants which we could divided in 3 themes. |
| Clarity of major themes | 31 | Were major themes clearly presented in the findings? | **Page 9-19,**  Presented three themes:  1) Dealing with chronic pain, 2) The current situation regarding aspects of Positive Health and 3) Future perspectives on health and quality of life. |
| Clarity of minor themes | 32 | Is there a description of diverse cases or discussion of minor themes? | **Page:20-22**  In the discussion we highlighted striking points from the results.  Diverse minor or major items are descript in the results**.** |

Developed from: Tong A, Sainsbury P, Craig J. Consolidated criteria for reporting qualitative research (COREQ): a 32-item checklist for interviews and focus groups. International Journal for Quality in Health Care. 2007. Volume 19, Number 6: pp. 349 – 357 Once you have completed this checklist, please save a copy and upload it as part of your submission. DO NOT include this checklist as part of the main manuscript document. It must be uploaded as a separate file.
